# Superhydrophobic-like tunable droplet bouncing on slippery liquid interfaces

**DOI:** 10.1038/ncomms8986

**Published:** 2015-08-07

**Authors:** Chonglei Hao, Jing Li, Yuan Liu, Xiaofeng Zhou, Yahua Liu, Rong Liu, Lufeng Che, Wenzhong Zhou, Dong Sun, Lawrence Li, Lei Xu, Zuankai Wang

**Affiliations:** 1 Department of Mechanical and Biomedical Engineering, City University of Hong Kong, Hong Kong 999077, China; 2 Department of Physics, Chinese University of Hong Kong, Hong Kong 999077, China; 3 Science and Technology on Microsystem Laboratory, Shanghai Institute of Microsystem and Information Technology, Chinese Academy of Sciences, 865 Changning Road, Shanghai 200050, China; 4 Shenzhen Research Institute, City University of Hong Kong, Shenzhen 518057, China

## Abstract

Droplet impacting on solid or liquid interfaces is a ubiquitous phenomenon in nature. Although complete rebound of droplets is widely observed on superhydrophobic surfaces, the bouncing of droplets on liquid is usually vulnerable due to easy collapse of entrapped air pocket underneath the impinging droplet. Here, we report a superhydrophobic-like bouncing regime on thin liquid film, characterized by the contact time, the spreading dynamics, and the restitution coefficient independent of underlying liquid film. Through experimental exploration and theoretical analysis, we demonstrate that the manifestation of such a superhydrophobic-like bouncing necessitates an intricate interplay between the Weber number, the thickness and viscosity of liquid film. Such insights allow us to tune the droplet behaviours in a well-controlled fashion. We anticipate that the combination of superhydrophobic-like bouncing with inherent advantages of emerging slippery liquid interfaces will find a wide range of applications.

## Abstract (web summary)

The impact of drops on surfaces is highly relevant to our daily life and many industrial applications, such as self-cleaning and ink printing. Here, Hao *et al*. show the transition from superhydrophobic-like drop bouncing, due to a trapped air layer, to substrate-dependent bouncing on a liquid thin film.

## Graphical abstract

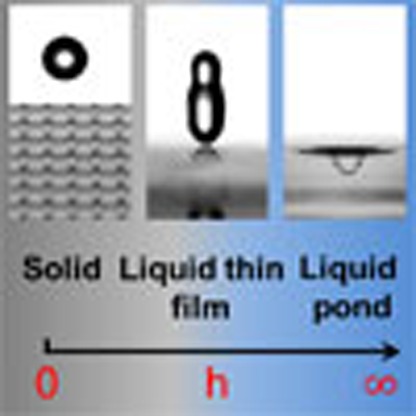

Droplet impact on liquid or solid surfaces has been extensively studied over the past century owing to its scientific importance and industrial applications in self-cleaning, anti-icing, spray cooling, inkjet printing, agriculture and forensic assay^1^,^2^,^3^,^4^,^5^,^6^,^7^,^8^,^9^,^10^,^11^,^12^,^13^,^14^,^15^,^16^,^17^. A wide range of experiments have demonstrated that impacting droplets can originate intriguing scenarios including rebound, splash, floating, pinning, coalescence and jetting, depending on the droplet itself, the air layer and the physiochemical properties of the substrate^18^,^19^,^20^,^21^,^22^,^23^,^24^,^25^,^26^,^27^,^28^. In particular, owing to small energy dissipation, droplet impinging on superhydrophobic solid substrates exhibits a spectacular complete rebound in a spherical or pancake shape^29^,^30^,^31^,^32^,^33^,^34^,^35^. Complete bouncing can also be induced at high temperatures by Leidenfrost effect^36^,^37^,^38^ or freezing temperatures by sublimation^39^.

On another research line, extensive progress has been made in the investigation of drop impact with another liquid body, either drop, bath or film^40^,^41^,^42^,^43^,^44^,^45^. Lord Rayleigh first reported the interactions between two colliding streams of droplets^46^. A droplet bouncing on a liquid surface has been elegantly shown to exhibit quantum-like properties, including double-slit interference, tunnelling and energy quantization^47^. Now, it is generally accepted that the air layer entrapped between the impinging droplet and underlying liquid plays an important role in modulating the outcome of droplet impact^41^,^47^,^48^, though it is susceptible to rupture at high-impacting velocity. To sustain a stable air layer and exert a lubrication force for bouncing promotion, external vibration has been used^49^,^50^. In most studies, the liquid layer is deep and the bouncing is limited to very low-impacting velocity. Recently, Gilet and Bush^51^ performed a comprehensive study on the bouncing, splitting and merging dynamics of a silicone oil droplet on an inclined thin oil film with very large viscosity. Under proper condition, the droplet was found to rebound completely. Similarly, de Ruiter *et al*.^52^,^53^,^54^ also reported that a liquid droplet even bounces off from the flat hydrophilic solid surface with the aid of thin air cushion, whose evolution during droplet impact was studied with the reflection interference contrast microscopy (RICM). It is interesting to treat the solid substrate as a thin liquid film with the viscosity close to infinity. Despite significant progress on the air layer entrapment dynamics, it remains elusive that how and to what extent the liquid substrate can modulate the bouncing behaviour of an impinging droplet.

In this work, we consider the situation of a water droplet impact on an immiscible liquid film with tunable thickness and viscosity. This situation is of relevance to many practical applications, especially those emerging applications boosted from the recent development of slippery liquid-infused porous surface^55^,^56^,^57^,^58^. Since there is an air layer entrapped on the thin liquid film, we can treat the film as composite interfaces. Such interfaces are in striking contrast to superhydrophobic solid surfaces. First, liquid interfaces are smooth whereas superhydrophobic solid surfaces are rough, with more ample areas for air entrapment. Second, different from the solid surfaces which are rigid and inhibit large droplet deformation in the direction normal to the substrate, the liquid interfaces are soft and mobile. Thus, it is expected that the impact dynamics of droplet on liquid interfaces will be totally different from that on superhydrophobic surfaces.

## Results

### Superhydrophobic-like bouncing

**Figure 1:**
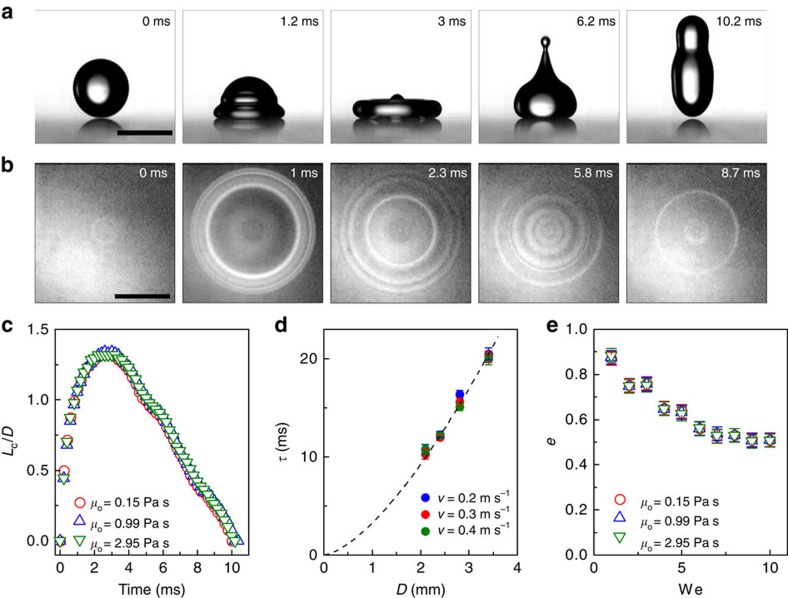
Superhydrophobic-like droplet bouncing. (**a**) Selected snapshots showing the complete rebound of droplet impacting on the composite interface (oil viscosity *μ*_o_=0.15 Pa s and thickness *h*=50 μm) under We=10. Scale bar, 2 mm. (**b**) Interference fringe patterns imaged by the RICM confirms the existence of a thin air layer during the entire impact process. Scale bar, 1 mm. (**c**) Time-resolved variation of droplet contact length *L*_c_ normalized by the droplet diameter *D*, that is, the spread factor *L*_c_/*D*, on the composite interfaces (*h*=50 μm) reveals that the spreading and retracting processes are independent of liquid viscosity. (**d**) Contact time of a bouncing droplet follows the scaling law *τ*∼(*ρD*^3^*γ*^−1^)^1/2^, which is consistent with theoretical analysis on superhydrophobic surfaces. (**e**) The variation of the restitution coefficient *e* as a function of We: *e* is independent of the oil viscosity *μ*_o_. The error bars for the contact time and restitution coefficient were obtained from the standard deviation of 10 sets of image data calculation.

Our experimental results are in striking contrast to the conventional perspective. We found that under proper impact conditions, a droplet impacting on composite interfaces exhibits a superhydrophobic-like complete rebound, or the bouncing behaviours are independent of the underlying liquid. Figure 1a shows selected snapshots of a ∼4.8 μl water droplet impacting on composite interfaces under a Weber number We=10, with We=*ρν*^2^*Dγ*^−1^. Here, *ρ* is the liquid density, *ν* is the impact velocity, *D* is the droplet diameter and *γ* is water surface tension. The droplet impact dynamics is recorded by a high-speed camera (Photron, FASTCAM SA4) with frame rate 5,000 fps. The viscosity of the oil in the composite interfaces is *μ*_o_=0.15 Pa s and the thickness is *h*=50 μm. Upon collision with the composite interfaces, the droplet spreads and displays a pancake shape at ∼3 ms. Then, the droplet moves inwards and finally bounces off the surface at ∼10.2 ms. Close inspection using RICM (wavelength *λ*=546 nm) coupled with high-speed camera reveals that there exists an interference fringe underneath the droplet throughout the bouncing process (Fig. 1b and Supplementary Movie 1), suggesting the presence of an intact air cushion. The air entrapment is also found on composite interfaces with larger *μ*_o_ (0.99 and 2.95 Pa s in our experiment).

We quantified the variation of droplet contact length *L*_c_ normalized by droplet diameter *D*, that is, the spread factor *L*_c_/*D*, for surfaces with different oil viscosity. As shown in Fig. 1c, all data collapse into a master curve, revealing that droplet spreading and retracting processes are irrespective of the viscosity of the underlying liquid substrate. The contact time *τ*, measured as the time during which the droplet is in apparent contact with the liquid substrate, follows the scaling law^59^ of *τ*∼(*ρD*^3^*γ*^−1^)^1/2^ with the fitting coefficient ∼0.875 (Fig. 1d), suggesting that *τ* is not disturbed by the soft interfaces as well. In Fig. 1e, we plot the variation of the restitution coefficient *e* as a function of We. Here, *e* is defined as the ratio of the droplet velocity after and before impact. For all samples with different viscosity, *e* decreases from ∼0.9 to 0.5 with increasing We. Taken together, water droplet impinging on thin liquid interfaces displays a signature reminiscent of elastic bouncing on superhydrophobic solid substrates and such a bouncing is different from those interesting work reported before^5^,^52^.

### Breakdown via air cushion rupture

**Figure 2:**
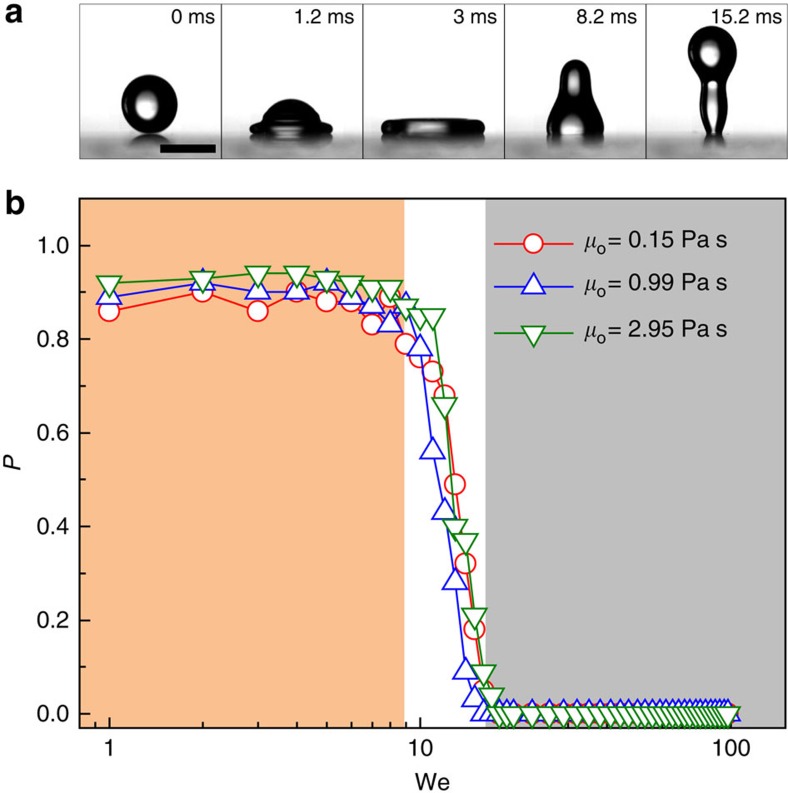
The breakdown of the superhydrophobic-like bouncing. (**a**) Selected time-lapsed images showing the impacting droplet on composite interfaces (*μ*_o_=0.15 Pa s and *h*=50 μm) at We=20. There is no bouncing observed owing to the rupture of air cushion. Scale bar, 2 mm. (**b**) The variation of the probability *P* of achieving complete rebound with air entrapment as a function of We on composite interfaces (*h*=50 μm). For We≲10, the percentage of complete rebound with air cushion entrapment is as high as 75–90%, whereas it drops down to nearly zero for We≳18.

The superhydrophobic-like bouncing on the thin liquid substrate requires the sustenance of a continuous and integral air cushion. Figure 2a presents selected time-lapsed images of a droplet hitting liquid film of *h*=50 μm and viscosity *μ*_o_=0.15 Pa s at We=20. As evidenced by RICM measurement, the air layer lasts <5 ms and then collapses across the whole interface (Supplementary Fig. 1 and Supplementary Movie 2). After that, the water droplet contacts the underlying oil phase and the bouncing is inhibited. To determine the critical We below which the air cushion would persist during the entire impact process, we analysed the probability *P* of achieving complete rebound without the rupture of air cushion. Figure 2b plots the variation of *P* as a function of We, in which each point is based on the average of 100 attempts. For We≲10, the percentage of complete rebound with an integral air layer entrapment is 75–90%, whereas it drops sharply to nearly zero for We≳18. Moreover, the oil viscosity has no apparent effect on the critical We, which is consistent with our experiment of droplet collision on pure oil bath with *h*=10 mm (Supplementary Fig. 2).

**Figure 3:**
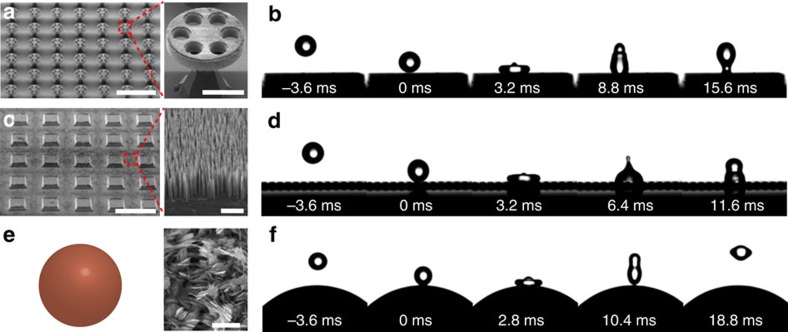
Liquid curvature effect on breakdown of superhydrophobic-like bouncing. (**a**) Scanning electron microscopic (SEM) image of as-fabricated circular mushroom post arrays (left panel, scale bar, 300 μm). The radius, centre-to-centre spacing, and height of mushroom post are 47, 200 and 85 μm, respectively. The entire surface was covered by silicon nanowire (right panel, scale bar, 50 μm) and infused with a thin layer of lubricating oil. (**b**) Selected snapshots showing the breakdown of superhydrophobic-like bouncing on oil-infused mushroom structure due to easy penetration of droplet into post arrays. (**c**) SEM image of silicon pyramid arrays with two-tier roughness (left panel, scale bar, 750 μm). The edge size, centre-to-centre spacing and height of pyramids are 300, 800 and 75 μm, respectively. The entire surface was decorated with silicon nanowire (right panel, scale bar, 50 μm) and infused with a thin layer of lubricating oil to form liquid curvature. (**d**) Selected snapshots showing the breakdown of the superhydrophobic-like bouncing on liquid-infused pyramid arrays. (**e**) Schematic diagram of as-fabricated copper ball (left panel) with a radius 10 mm decorated with CuO nanoblade (right panel, scale bar, 2 μm) for oil infusion. (**f**) Selected snapshots showing the droplet impacting on liquid-infused spherical surface. The droplet bouncing was found to be close to that on flat thin film.

Interestingly, we found that the breakdown of the superhydrophobic-like bouncing was also sensitive to the liquid curvature. To evaluate how the curvature of liquid affects the droplet impact, we further prepared three control surfaces with two-tier roughness. The first surface was mushroom post arrays, with the radius, centre-to-centre spacing and height of posts being 47, 200 and 85 μm, respectively (Fig. 3a). The second surface was silicon micro-pyramid arrays, with the edge size, centre-to-centre spacing and height of pyramids being 300, 800 and 75 μm, respectively (Fig. 3c). The third surface was the copper ball with a radius of 10 mm (Fig. 3e). All the as-prepared samples were covered with nanostructures formed by wet or dry etching (See Methods section), exhibiting a superhydrophobic property after the hydrophobic treatment. To form a stable liquid film with different curvature, we then infused lubricating liquid onto as-prepared surfaces. Thus, these surfaces can be treated as liquid films with radii of curvature ranging between ∼50 and 10 mm. Upon collision with liquid films with smaller radii of curvature (Fig. 3b,d), the droplet finally adhered to liquid films after spreading, probably due to its penetration into liquid post arrays (Supplementary Movies 3 and 4). In contrast, the droplet hitting the large ball displayed a complete rebound, which was close to the superhydrophobic-like bouncing as observed on flat thin liquid film (Fig. 3f and Supplementary Movie 5). This is expected, since the surface with such a large radius of curvature is close to flat liquid film and the effect of liquid curvature on droplet bouncing is gradually suppressed.

### Substrate-dependent bouncing

**Figure 4:**
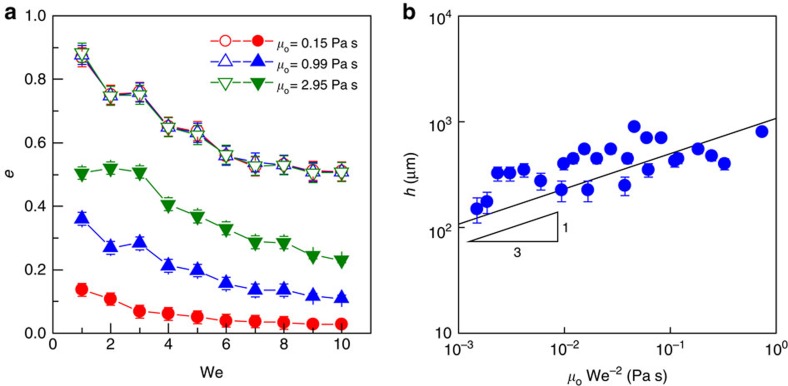
Energy analysis. (**a**) The variation of the restitution coefficient *e* of droplet impact on oil bath (*h*=10 mm) under different We and *μ*_o_. It is clear that compared to superhydrophobic-like bouncing, the bouncing on the liquid bath is substrate dependent, with a strong dependence of the restitution coefficient *e* on the oil viscosity. The small *e* for the droplet impacting on the liquid bath is due to the large viscous energy dissipation as a result of a pronounced deformation at the liquid/air interfaces. (**b**) The plot of the critical thickness as a function of We and *μ*_o_, under which the impinging droplet exhibits superhdyrophobic-like bouncing behaviour and follows the power law scaling of *h*_c_∼(*μ*_o_*We*^−2^)^1/3^. The error bars for restitution coefficient and critical thickness were obtained from the standard deviation of 10 sets of image data calculation.

We found that the droplet rebound dynamics on oil bath (*h*≫*D*) is totally different from that on the thin oil film. Figure 4a illustrates the variation of *e* for a droplet impinging on a deep oil bath (*h*=10 mm) and thin film (*h*=50 μm), respectively. In all cases, the air layer is maintained during the entire impact process, as evidenced by our optical measurement. The droplet bouncing on oil bath exhibits a substrate-dependent signature, with its restitution coefficient *e* strongly dependent on oil viscosity. Moreover, compared with the superhydrophobic-like bouncing, there is a marked reduction in *e* under the same We, suggesting that the droplet bouncing is dramatically modulated by liquid substrate. Careful inspection reveals that the radius of the hemispherical cavity formed in oil bath during impact is comparable to the droplet size, whereas there is almost no marked deformation observed in the thin composite film. To validate this observation, we then systematically performed droplet impact experiments on oil films with three different viscosities under different We. The thickness of oil films ranges from 50 to 1,000 μm with a step increment of 50 μm. As an example, in Supplementary Fig. 3 we plot the restitution coefficient as a function of *h* for oil films with viscosity of 0.15, 0.99 and 2.95 Pa s, respectively. It is obvious that the restitution coefficient *e* is gradually decreased with increasing oil thickness *h*, though the amplitude of the decrease is influenced by the oil viscosity *μ*_o_. To identify the critical thickness *h*_c_ to distinguish the superhydrophobic-like and substrate-dependent bouncing, we chose the restitution coefficient obtained on thin film with thickness *h*=50 μm as the baseline. Also, we deemed a droplet bouncing as superhydrophobic like given that its restitution coefficient was within 5% deviation from the baseline, that is, (*e*–*e*_o_)/*e*_o_≤5%, where *e*_o_ was the restitution coefficient for the sample with *h*=50 μm. Supplementary Figure S3b illustrates the phase diagram showing how the interplay of oil thickness *h* and viscosity *μ*_o_ affects the bouncing outcome at We=2. The superphydrophobic-like bouncing is reflected by open symbols while solid symbols denote the substrate-dependent bouncing regime. The droplet bouncing is independent of the substrate when the liquid film is below the critical thickness. By contrast, for a liquid film with adequate thickness, the droplet bouncing is transformed into substrate-dependent regime. Under a fixed We, the critical thickness *h*_c_ distinguishing the superhydrophobic-like (substrate independent) and substrate-dependent bouncing regimes is found to be dependent on the liquid viscosity. The critical thickness *h*_c_ for the oil film with viscosity 0.15, 0.99 and 2.95 Pa s at We=2 is ∼250, 400, and 800 μm, respectively.

We conducted a simple energy analysis to elucidate the dependence of the droplet bouncing on the intricate interplay between the oil viscosity and thickness of liquid substrate as well as the Weber number. The total energy dissipation of an impacting droplet on a slippery surface with *h* can be expressed by:


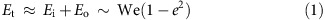


where *E*_i_ and *E*_o_ represent the energy dissipation caused by the water internal flow and oil flow, respectively. Note that here in our analysis we neglect the energy dissipation in air flow since the thickness of air film ranges from hundreds of nanometres to a few micrometres^51^,^52^. Based on the lubrication approximation theory, the energy dissipation in the oil film *E*_o_ can be estimated by:


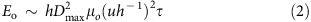


where *u*∼*ρν*^2^*h*^2^(*D*_max_*μ*_o_)^−1^ is the characteristic velocity of the oil film and *τ*∼(*ρD*^3^*γ*^−1^)^1/2^ is the contact time. Substituting *τ* into *E*_o_ and combining with equation (1), we have


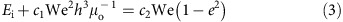


where *c*_1_ and *c*_2_ are pre-factors. The internal energy dissipation *E*_i_ in the superhydrophobic-like bouncing can be approximated as 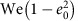. Thus, *h*_c_ in the case of superhydrophobic-like bouncing is expressed as:


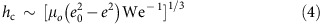


Moreover, a bouncing droplet of non-viscous fluid can be modelled as a spring^31^, and the restitution coefficient *e* follows the power law of We^−1/2^. Thus, equation (4) can be scaled as *h*_c_∼(*μ*_o_We^−2^)^1/3^. This scaling is in good agreement with our experimental data as shown in Fig. 4b.

## Discussion

**Figure 5:**
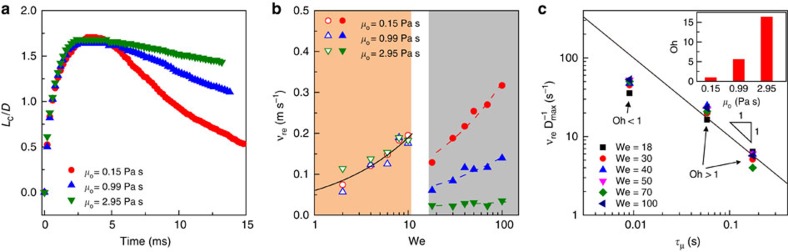
Substrate-dependent retraction dynamics. (**a**) Comparison of time-resolved evolution of the spread factor *L*_c_/*D* on samples of *h*=50 μm with different viscosity. The We is 20. The values of *L*_c_/*D* in the retraction stage are dependent on the oil viscosity, displaying a substrate-dependent retraction behaviour. (**b**) Calculated retraction velocity *ν*_re_ as a function of We indicating that the retraction velocity is independent of the oil viscosity in the superhydrophobic-like bouncing regime, that is, *We*≲10. By contrast, with the rupture of thin air cushion, that is, *We*≳18, the retraction velocity becomes highly dependent on the oil viscosity. (**c**) The droplet retraction rate *ν*_re_/*D*_max_ as a function of viscous time scale *τ*_*μ*_. The measured retraction rates 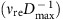 overlap well with the scaling of 
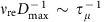
, where 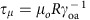
is the viscous time scale (corresponding to two datasets with Oh>1). For Oh≈0.86 (*μ*_o_=0.15 Pa s), the retraction rates diverge from the master curve because the inertia force and viscous force are comparable in this case.

To elucidate how the collapse of air layer or the breakdown of the superhydrophobic-like bouncing affects droplet retraction dynamics, we compared the spread factor on samples with different *μ*_o_ as a function of time at We=20. As shown in Fig. 5a, the contact line dynamics in the spreading stage is nearly the same, while it becomes closely reliant on the oil viscosity in the retraction stage, suggesting a substrate-dependent retraction behaviour. This is as evidenced by the contact line retraction velocity *ν*_re_ measurement as shown in Fig. 5b. To analyze the effect of oil viscosity on droplet retraction dynamics, we calculated the Ohnesorge number Oh=*μ*_o_(*ρRγ*_oa_)^−1/2^ which measures the relative importance of inertia, viscous and surface tension forces, where *γ*_oa_ is the surface energy of oil and *R* is the radius of water droplet. Since Oh for liquid interfaces with *μ*_o_=0.99 and 2.95 Pa s is 5.52 and ∼16.31 (Fig. 5c inset), respectively, the droplet retraction process is primarily dominated by the viscous force. Indeed, measured retraction rates 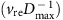 overlap well with the scaling of 
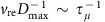
, where 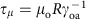
is the viscous time scale^60^. However, for Oh≈0.86 (*μ*_o_=0.15 Pa s, Fig. 5c inset), the retraction rates diverge from the master curve because the inertia force and viscous force are comparable in this case.

**Figure 6:**
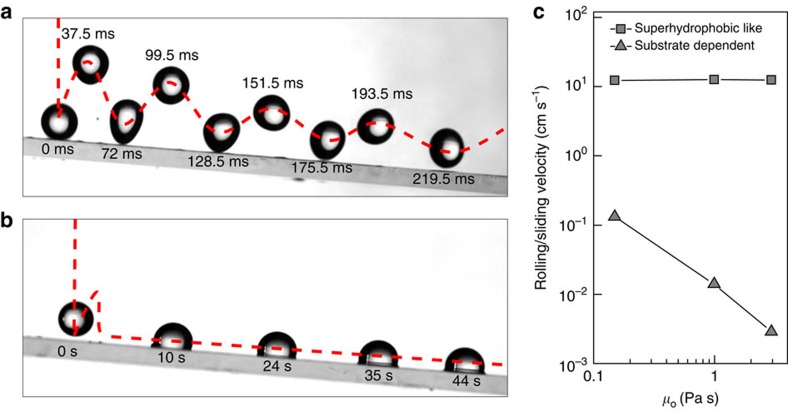
Fast droplet shedding assisted by superhydrophobic-like bouncing. (**a**) Time-lapsed images of water droplet collision on the composite interfaces under a tilt angle of ∼5°. The droplet undergoes repetitive rebounding and falling for several cycles with trajectories indicated by red dash line and finally rolls off the surfaces within ∼230 ms. (**b**) The sliding dynamics of droplet in the substrate-dependent regime, with a sliding velocity two order of magnitude smaller than that on superhydrophobic-like bouncing regime. (**c**) Comparison of the calculated droplet rolling/sliding velocity as a function of liquid viscosity.

Finally, we demonstrated the utility of superhydrophobic-like bouncing for fast droplet shedding. Figure 6a shows time-lapsed images of water droplet collision on the composite interfaces under a tilt angle of ∼5° (Supplementary Movies 6 and 7). To make the oil film with *h*=50 μm stable when it is tilted, the oil is locked in a thin porous Teflon membrane with thickness ∼20 μm. The droplet undergoes repetitive rebounding and falling for several cycles with trajectories indicated by dashed red line and finally rolls off the surfaces within ∼230 ms. In contrast, the droplet in the substrate-dependent retraction regime (large We) keeps intimate contact with underlying liquid and the sliding velocity is at least two orders of magnitude smaller than that in the superhydrophobic-like bouncing on the thin oil film (Fig. 6b,c). The enhanced droplet bouncing/sliding in the superhydrophobic-like bouncing regime is ascribed to the smooth nature of the composite interfaces, which helps to maintain a lubricating air layer even without the aid of external vibration. Thus, our results illustrate the important and previously unexplored effect of liquid substrate on the droplet impact. We expect that the occurrence of unexpected superhydrophobic-like bouncing and fast droplet removal on emerging slippery surfaces will extend their wide applicability in many processes^55^,^56^.

## Methods

### Slippery liquid film preparation

The thin oil film was formed by directly pouring into the transparent glass vessel, and the thickness of oil film is precisely controlled by the oil volume. The glass surface was thoroughly cleaned and plasma treated to remove all dust particles before the experiment of drop impact. In our experiment, the oil used for the experiment is perfluorinated fluid Dupont Krytox 103, 105 and 107 with *μ*_o_=0.15, 0.99 and 2.95 Pa s, respectively. To make the liquid film stable when it is tilted, the lubricating oil is locked in a thin porous Teflon membrane with thickness ∼20 μm. These Teflon membranes were purchased from the Sterlitech Corporation and used as received without further modification.

### Fabrication of silicon-based superhydrophobic surfaces

The silicon nanowires and pyramid arrays were fabricated based on 425-μm thick silicon wafer. For the fabrication of pyramid arrays, we first conducted wet anisotropic etching in the KOH solution. The concentration of KOH solution was 40 wt% and the etching temperature was 40 °C. Then, the deep reactive ion etching (DRIE) process was used to produce nanowire arrays. The DRIE process included cyclic passivation and etching modes in which C_4_F_8_ and SF_6_ were used. The coil power was set at ∼600 W. In the etching cycle, the SF_6_ flow rate was ∼130 sccm and platen power was set at ∼12 W. In the passivation cycle, the C_4_F_8_ flow rate was ∼85 sccm. After the etching, the entire wafer was immersed into the piranha solution (3:1 mixture of H_2_SO_4_ and H_2_O_2_ at 120 °C) for 10 min to remove the polymer deposited on the surface. The mushroom post arrays were fabricated based on the SOI silicon wafer. For the fabrication of mushroom post arrays, both wet etching and DRIE were implemented to form complex post arrays. Note that the nanowire arrays covered on the entire surface were formed through the metal-assisted chemical etching. Briefly, the mushroom post arrays were immersed in an etchant solution (HF=5 mol l^−1^ and AgNO_3_=0.005 mol l^−1^) for 30 s to form Ag particles on the surface. After rinsing in deionized (DI) water, the sample was immediately transferred into another solution (HF=5 mol l^−1^ and H_2_O_2_=0.3 mol l^−1^) for 60 s at 50 °C to form Si nanowires on the entire surface. After removing deposited Ag dendrites from the surface in 20% HNO_3_, the sample was rinsed with DI water and blow-dried with N_2_.

### Fabrication of CuO nanoblade

The commercially available copper ball with a radius 10 mm was used to form large liquid curvature. The copper ball was first ultrasonically cleaned with acetone, ethanol and water for 20 min, respectively, and then immersed in 1 M hydrochloric acid solution for 30 s to remove the native oxide layer on the surface. To create CuO nanostructures, the as-prepared copper ball was placed into the alkaline solution of NaOH, NaClO_2_, Na_3_PO_4_·12H_2_O and water with weight percent: 5, 3.75, 10 and 100%, respectively, and the temperature was maintained at 95 °C for 10 min. After oxidation, the blade-like CuO nanostructure was formed with thickness of ∼2 μm. Finally, the as-prepared surface was immersed in 1 mM hexane solution of trichloro(1H,1H,2H,2H-perfluorooctyl)silane for about one hour, followed by heat treatment at ∼150 °C for 30 min to render the surface superhydrophobic.

### Droplet impact experiment

The water droplet with fixed volume was produced from a fine stainless steel needle connected to a syringe pump (KD Scientific) from a pre-determined height, and the droplet impact dynamics was recorded by a high-speed camera (Photron, Fastcam SA4) with frame rate 5,000 fps and analysed with software ImageJ. All the experiments were conducted in ambient environment with room temperature and relative humidity ∼50%.

### Air layer visualization using the interference fringe

The images of interference fringe of the thin air film during impact were observed by RICM with wavelength *λ*=546 nm through the transparent substrate and recorded with the high-speed camera (Photron, Fastcam SA4) at frame rate 10,000 fps and shutter speed 1/15,000 s. The experiment was conducted under the ambient condition. The images acquired were analysed using software ImageJ.

## Additional information

**How to cite this article:** Hao, C. *et al*. Superhydrophobic-like tunable droplet bouncing on slippery liquid interfaces. *Nat. Commun.* 6:7986 doi: 10.1038/ncomms8986 (2015).

## Supplementary Material

Supplementary Information Supplementary Figures 1-3

Supplementary Movie 1 Bottom visualization of the impact dynamics of a water drop of ~ 4.8 μL hitting on the compound interfaces with oil viscosity *μ*o = 0.15 Pa·s proving the existence of a robust air film during the entire process. The Weber number *We* is 10.0.

Supplementary Movie 2 RICM visualization of interference fringe from the bottom view demonstrating the rupture of the thin air film within ~ 5 ms upon collision on the oil film at *We* = 20.0. The rupture point (indicated by the white arrow) is randomly distributed during different impact experiments.

Supplementary Movie 3 Dynamics of droplet impacting on the mushroom post arrays infused with thin liquid film, demonstrating the rupture of air cushion and breakdown of the superhydrophobic-like bouncing. The Weber number is 8.0.

Supplementary Movie 4 Dynamics of droplet impacting on the silicon micro-pyramid arrays infused with a thin liquid film, demonstrating the rupture of air cushion and breakdown of the superhydrophobic-like bouncing. The Weber number is 8.0.

Supplementary Movie 5 Dynamics of droplet impacting on the Cu ball infused with a thin liquid film, demonstrating the complete bouncing of droplet. The Weber number is 8.0.

Supplementary Movie 6 A time-lapsed video showing cyclic impact and bouncing of a water droplet on an inclined thin oil film at *We* = 5.0. The air layer is sustained during the entire process.

Supplementary Movie 7 Superhydrophobic-like bouncing of continuous water droplets at *We* = 5.0 from an inclined thin oil film.
