# Supplementary material for: Superhydrophobic-like tunable droplet bouncing on slippery liquid interfaces
Source: Nat Commun. 2015 Aug 7;6:7986. doi: 10.1038/ncomms8986 (PMC4918357; doi:10.1038/ncomms8986)
Supplement: Supplementary Information — Supplementary Figures 1-3 [file ncomms8986-s1.pdf]

Supplementary Figures

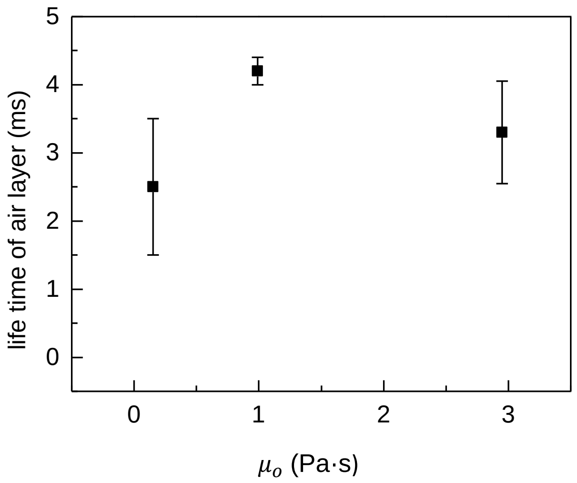

Supplementary Figure 1 | The variation of the life time of thin air layer on liquid substrates of different viscosity. The thickness of the liquid substrate is h=50 μm and the Weber number is We = 20.

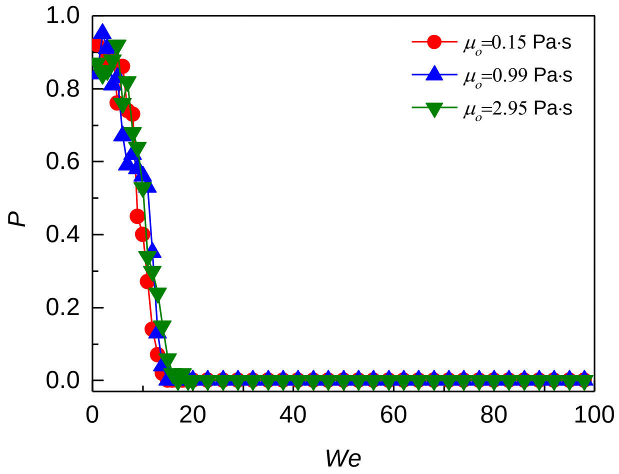

Supplementary Figure 2 | The probability (P) of sustaining an intact thin air cushion on pure oil bath under different We. For $We \lesssim 10$, the percentage of achieving complete rebound with an integral air layer entrapment is 75% ~ 90%, whereas it drops sharply to nearly zero for $We \gtrsim 18$. Moreover, the oil viscosity has no apparent effect on the probability of sustaining an intact thin air cushion.

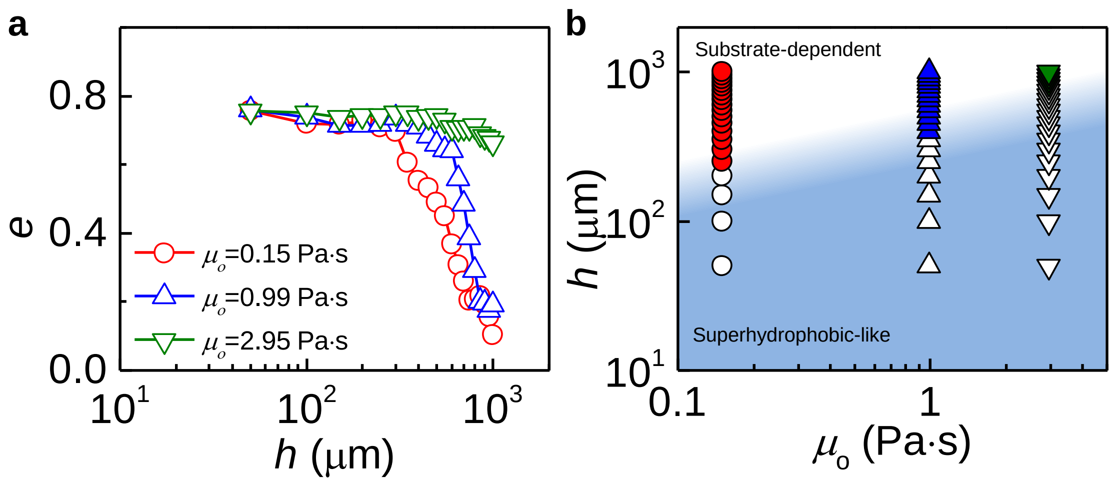

Supplementary Figure 3 | (**a**) The variation of the restitution coefficient *e* as a function of oil thickness *h* for oil films with viscosity of 0.15 Pa·s, 0.99 Pa·s and 2.95 Pa·s, respectively. The *We* is 2.0. (**b**) The dependence of superhydrophobic-like bouncing (open symbols) and substrate-dependent bouncing (solid symbols) on *h* and μ_o. In the superhydrophobic-like bouncing regime, the effect of liquid substrate on the bouncing is negligible whereas *e* is greatly affected by the oil film in the substrate-dependent regime.
